# Spatial transcriptomic mapping of early immune responses to an adjuvanted recombinant hemagglutinin vaccine

**DOI:** 10.3389/fimmu.2026.1831403

**Published:** 2026-07-24

**Authors:** Laurelle Jackson, Sarah M. Williams, James Sinclair, Ana Kume, Azadeh Bahadoran, Ingeborg M. Langohr, Hillary Danz, Bin Lu, Dinesh S. Bangari, Amanda J. Cox, Laurence Macia, Sudha Chivukula, Nicholas P. West

**Affiliations:** 1 School of Pharmacy and Medical Science and Central Facility for Genomics, Griffith University, Gold Coast, QLD, Australia; 2 Institute of Biomedicine and Glycomics, Griffith University, Gold Coast, QLD, Australia; 3 The Queensland Cyber Infrastructure Foundation (QCIF), The Precinct, Fortitude Valley, Brisbane, QLD, Australia; 4 mRNA Center of Excellence, Sanofi, Waltham, MA, United States; 5 Global Discovery Pathology and Multimodel Imaging, Sanofi, Cambridge, MA, United States; 6 Global Clinical Development Sanofi, Woolloongabba, QLD, Australia

**Keywords:** immune response, lymph nodes, site of injection, spatial profiling, vaccines

## Abstract

Vaccination relies on innate and adaptive responses initiated at the injection site followed by activation in draining lymph nodes. However, high-plex, spatially resolved maps that couple injection site signals with transcriptomic programs in draining lymph nodes over time are still lacking. Here, we used spatial transcriptomics to map early responses to an adjuvanted recombinant haemagglutinin (rHA) protein vaccine formulated with Sanofi’s proprietary oil-in-water adjuvant AF03. Mice received intramuscular rHA-protein or PBS. Injection site muscle and draining lymph nodes were collected at Days 1 and 7 post-prime for GeoMx™ Digital Spatial Profiling (DSP) and CosMx™ Spatial Molecular Imaging (SMI) single-cell imaging. In lymph nodes, rHA-protein vaccination induced early innate pathway activation in non-follicular regions at Day 1. By Day 7, adaptive pathways were enriched within follicles, with differentially expressed genes (DEGs) linked to germinal center (GC) formation, transcriptional regulation and signaling. These changes coincided with expansion of a proliferative GC-like B-cell cluster within the follicular area. At the injection site, 20x20 µM spatial grids were used rather than segmented cells. Clusters, enriched for fibroblast and muscle cells showed Day 1 induction of chemokines and interferon stimulated genes, concentrated in fibroblast rich stromal layers. These spatially organized tissue programs were accompanied by transient systemic cytokine elevations and increased antigen-specific IgG titers. Together, these data provide a previously unresolved view of what, when and where local inflammation and lymph node organization are spatially and temporally associated during early responses following vaccination with an adjuvanted rHA-protein vaccine.

## 1 Introduction

Vaccination remains one of the most effective public health interventions, relying on the orchestration of innate and adaptive immune responses to generate robust and long-lived protection. This cascade begins at the injection site, typically intramuscular, where the injection of antigen and adjuvant trigger chemokine and cytokine production by resident stromal and immune cells, recruiting neutrophils, monocytes dendritic cells (DCs). Antigen presenting cells and soluble antigen then reach the draining lymph node, where antigen presentation, T and B cell activation, and GC formation occur (1–7). Within the lymph node, structure dictates function. Spatially distinct compartments, including the subcapsular sinus, T cell zone (paracortex), B cell follicles and interfollicular regions, support specialized functions in antigen capture and presentation, lymphocyte activation and the generation of adaptive immune responses respectively. Effective immunity therefore depends not only on the magnitude of the response, but also on the timing and anatomical localization of inflammatory and immune signals across the injection site and these distinct lymph node compartments. Together, these spatiotemporal patterns may contribute to vaccine efficacy, tolerability and reactogenicity.

However, high-plex, spatially resolved maps that couple vaccine injection site signals with transcriptomic profiles across intact, non-dissociated draining lymph nodes over time are still lacking. Indeed, adjuvanted protein or subunit vaccines have been shown to induce coordinated immune responses that span the injection site and draining lymph node. In these formulations, the adjuvant component promotes early local innate activation, including chemokine induction, recruitment of neutrophils and monocytes, antigen uptake and transport of antigen-loaded cells to the draining lymph node (2, 8–14). Within the draining lymph node, antigen presentation supports antigen-specific T cell activation, B cell activation, germinal center formation, antibody production and the development of immune memory following adjuvanted protein or subunit vaccination (14–18). These responses have generally been studied using separate cellular, histological and transcriptomic approaches, rather than integrated spatial profiling of intact tissues. Recent advances in high-plex spatial transcriptomics make it possible to map these responses within intact tissues, helping to define what immune profiles emerge, when they arise and where they are spatially organized across the injection site and draining lymph node during early vaccine responses.

Here, GeoMx™ DSP and CosMx™ SMI was utilized to profile early responses to an adjuvanted rHA-protein vaccine with the oil-in-water emulsion adjuvant AF03. Murine injection site muscle and draining lymph nodes at Days 1 and 7 post-prime were analyzed, focusing on cell cluster responses at the site of injection and compartment-resolved dynamics within the lymph nodes. We found that early innate activation in muscle was associated with coordinated lymph node signaling, prominent in non-follicular regions at Day 1 and within follicles by Day 7, coinciding with proliferating GC-like B cells. At the site of injection, cytokine/chemokine expression localized to fibroblast-rich regions and in the lymph nodes, responses were follicular-centered responses rather than diffuse extrafollicular activation. Together, these results provide foundational, spatially resolved insights for an AF03 adjuvanted recombinant protein vaccine that may guide ongoing efforts to optimize vaccine efficacy and design.

## 2 Materials and methods

### 2.1 Ethics statement

All experimental procedures involving animals were conducted in accordance with the guidelines established by the US National Institutes of Health and were approved by the Institutional Animal Care and Use Committees (IACUC) at Sanofi (IACUC AUP Number 21/500). Housing and handling of animals were performed in accordance and compliant with the standards of the Association for Assessment and Accreditation of Laboratory Animal Care, the Animal Welfare Act as amended, and the Public Health Service Policy.

### 2.2 *In vivo* studies

For immunogenicity studies, groups of 3 female BALB/c mice (Mus musculus) per treatment group were immunized under isoflurane anesthesia with a dose of 0.05 mL of rHA-protein (Sanofi, in-house) adjuvanted with AF03 (Sanofi, in-house) or PBS diluent via the IM route in the quadriceps, on Day 0 and 21 as described in Danz et al., (16). Given the high cost, substantial imaging time, complex sample preparation and data analysis requirements associated with high-plex spatial profiling, GeoMx™ DSP and CosMx™ SMI analyses were restricted to a subset of animals (n=3 per group/timepoint) within the available study budget.

**Figure 1:**
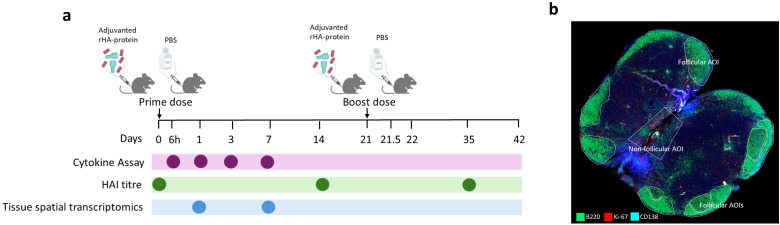
Experimental and sample preparations used in this study. **(a)** experimental design and timeline. **(b)** representative region of interest (ROI) selection on the GeoMx™ DSP. Segments were collected based on morphology marker staining (B220 segment – B cells). Non-segmented ROIs were based on region, either non-follicular or follicular regions. An *n* = 3 for biological replicates for each vaccine and each timepoint of lymph node collection. **(A)** Source: Created by the authors using BioRender.com under a BioRender publication license.

Approximately 200 *μ*L of blood was collected in SST tubes from mice via submandibular or orbital sinus bleeds on study Days 0, 14 and 35 from all animals under sedation (**Figure 1a** depicts times that samples were collected). Blood samples were allowed to clot for a minimum of 30 minutes at room temperature before centrifugation at 1000–1300 g for 5–10 minutes. Recovered sera was divided into 2 cryovials and stored at −80 °C.

### 2.3 Tissue processing and collection

On day 1 and 7 post-administration, the mice treated with either PBS or adjuvanted rHA-protein had injection site muscle and draining lymph nodes collected in 10% neutral buffered formalin (NBF) and then paraffin-embedded (FFPE) after a minimum of 48 hours in NBF. Three 5 µm tissue sections were cut from each FFPE muscle and lymph node block and mounted onto charged slides, ensuring placement within the maximum allowable scanned area for both the GeoMx™ DSP and CosMx™™ SMI platforms. One slide was hematoxylin and eosin-stained (H&E) and digitally scanned for assessment by a pathologist (**Supplementary Figures 1a, b**).

### 2.4 GeoMx™ DSP workflow

Details of the GeoMx™ DSP workflow were recommended by NanoString Technologies, (Seattle, WA, USA). Briefly, the workflow consisted of five major steps; 1) de-paraffinization (1 h at 65 °C), 2) antigen retrieval (ER2 for 20 min at 100 °C) and proteinase K treatment (0.7 *µ*g/ml for 10 min at 37 °C) using the Leica BOND RX (Leica Biosystems, Wetzlar Germany), 3) incubation with GeoMx™ DSP Mouse Whole Transcriptome Atlas probes, morphology markers and visualization antibodies, 4) Region of interest (ROI) placement guided by morphology marker staining, ultraviolet exposure to release and capture probe barcodes from ROIs and 5) sequencing of ROIs. Panel markers for the lymph node included B220 (532 channel, Thermo Fischer Scientific, Scoresby, VIC, Australia), CD138 (594 channel, Thermo Fischer Scientific, Scoresby, VIC, Australia), Ki-67 (647 channel, Abcam, Melbourne, VIC, Australia). Syto13 (532 channel, Seattle, WA, USA) was used as a nuclear stain. The study included 51 and 89 ROIs from the follicular and non-follicular regions respectively. Captured oligo barcodes from the ROIs were collected in 96-well plates, sealed and stored at -80 °C for sequencing. Sequencing was performed by the Australian Genome Research Foundation (AGRF). Sequencing libraries were sequenced on the Illumina NovaSeq 6000 following sequencing library generation using indices. Fastq files were processed through the NGS pipeline and converted to DCC files for downstream analysis.

### 2.5 GeoMx™ DSP ROI selection

For the GeoMx™ DSP assay, fluorescently labeled anti-B220, anti-CD138, and anti-Ki-67 antibodies were used to identify B cells, plasma cells, and proliferating cells, respectively (**Figure 1b**). Whole regions of interest (ROIs) were selected from the morphology images with guidance from H&E images (**Supplementary Figure 1a**). ROI selection was performed blinded to treatment group and timepoint. Follicular ROIs were defined as well-demarcated regions with dense B220 staining, consistent with B cell follicular areas. Where possible, follicular ROIs were placed centrally within B220-rich regions and avoided tissue folds, areas of poor staining or regions with obvious tissue disruption were avoided. The remaining lymph node regions, including the cortex, medulla and subcapsular sinus, were characterized by less dense or more diffuse B220 staining. Due to their overlapping, continuous architecture, together with limitations in AOI size and the minimum number of cells required for collection within each AOI, these regions frequently overlapped within individual AOIs and were therefore collectively grouped as non-follicular regions. This ROI selection criteria were performed across all mice, treatment groups and timepoints to maintain consistency between samples. Comparable ROI placement and size were used wherever possible as well as ensuring that each ROI met the GeoMx collection requirements for tissue area of sufficient cell/nuclear content (≥100 nuclei/cells per ROI).

### 2.6 GeoMx™ DSP quality control, normalization and data analysis

The GeoMx™ DSP data analysis suite was used for data processing, quality control, and target filtering. Segment QC and BioProbe QC modules were applied with default parameters, and ROIs with <10,000 deduplicated reads were excluded. For each ROI, the limit of quantitation (LOQ) was defined as the geometric mean + 2 geometric SDs of negative-control probes, and targets detected above the LOQ in ≥95% of samples were retained. The dataset was imported into R (v4.5.1) as a spatial expression object using standR (v1.13.0) and normalized with the Remove Unwanted Variation (RUV) method to correct for library-size and compositional bias. Differential expression was assessed with limma (19), considering genes with FDR < 0.05 significant. Because multiple ROIs originated from the same mouse, duplicate-correlation in limma was used to account for within-mouse correlation and avoid pseudoreplication. Functional enrichment was performed by Gene Set Enrichment Analysis (GSEA) using Reactome pathways. Significantly enriched terms (FDR < 0.05) were grouped into immune system, signal transduction, disease, and cell-cycle categories.

### 2.7 CosMx™ SMI workflow

Details of the CosMx™ SMI workflow were as recommended by NanoString Technologies, (Seattle, WA, USA). Briefly, the workflow consists of five major steps; 1) de-paraffinization (60 °C, overnight), 2) antigen retrieval and proteinase K treatment on the Leica BOND RX (rehydration, ER1 antigen retrieval, 10 min at 100 °C; proteinase K, 1 *µ*g/mL for 10 min at 37 °C), 4) Fiducials (0.001% in 2×SSC/0.001% Tween-20) were then applied, 5) fixation (10% neutral-buffered formalin), and 6) blocking (100 mM NHS-acetate). Sections were hybridized overnight at 37 °C with the CosMx™ SMI Mouse Universal Cell Characterization 1K RNA panel, then subjected to stringent washes (50% formamide in 2×SSC at 37 °C). Prior to flow-cell assembly, nuclei (DAPI) and cells were stained using the CosMx™ Universal Cell Segmentation kit (RNA; CD298, B2M, PanCK, CD45) plus CD3. Following flow cell assembly, the flow cells were loaded onto the CosMx™ SMI and a preview scan of the entire flow cell was taken, where the entire tissue for each tissue section was gridded with fields of view (FOVs) Thereafter, sequencing on the CosMx™ SMI platform was performed through iterative cycles of reporter probe hybridization, high-resolution imaging, and fluorescent signal cleavage.

### 2.8 CosMx™ SMI quality control, normalization and data analysis

CosMx™ data were analyzed in R using the SpatialFeatureExperiment toolkit (20). Cells with <50 transcripts or >0.25% negative-control content were excluded and counts were normalized by library size. Dimensional reduction was performed on the 30% most variable genes (blocked by tissue sample) using the top 30 principal components for UMAP and Louvain clustering (k = 30) with scran and scater packages (21, 22). Cell segmentation used the *cyto2* model in Atomx, which more accurately identified densely packed GC cells than the default cellpose model (**Supplementary Figure 1c**). Due to difficulty acquiring acceptable cell segmentation on the site of injection samples, sections were gridded into 20x20 µM grid squares rather than true cells (**Supplementary Figure 1d**). Representative FOVs of lymph node and site of injection tissues samples along with associated cell types defined post-data analysis. Cells with less than 50 transcripts or more than 0.25% negative control content were removed. Counts were normalised by library size. Dimensional reduction performed on the 30% most highly variable genes blocked on tissue sample, and the top 30 principal components, used for UMAP and clustering (Louvain method, k=30), using scran and scater packages (22, 23). Automatic cell typing was performed with SingleR (24) using reference datasets from Kim et al., (7) and Cui et al., (25). Cluster labels were refined manually based on marker-gene enrichment, with subclustering where transcriptional diversity was warranted. For marker gene analysis, data were subsampled to 1,000 cells per cluster. Cell-type proportion differences between treatment groups and time points were tested using transformed proportions via the propeller method in speckle package (19, 26). Differential expression per cluster was calculated by pseudobulk analysis with limma (19), including only clusters with ≥50 cells and ≥100 transcripts per cell. Pathway enrichment for each contrast was performed using FGSEA (25) and Reactome annotations. Enriched pathways (FDR < 0.05) were grouped into immune system, signal transduction, RNA metabolism, and gene expression categories.

### 2.9 Luminex cytokine assay

Multiplex cytokine levels were quantified using a 32 plex Mouse Cytokine/Chemokine Magnetic Bead Panel (MCYTOMAG-70K, Millipore). Standards and samples were prepared according to the manufacturer’s instructions. Data were acquired on a Luminex™ xMAP INTELLIFLEX™ system using bead regions defined in the protocol and analyzed using Belysa Curve Fitting Software (Sigma Aldrich). Standard curves were generated, and sample concentrations were calculated in pg/mL.

### 2.10 Enzyme linked Immunosorbent assay for recombinant HA-specific IgG

Pierce Copper Coated high binding capacity (Nunc 96 wells plates Thermo Fisher, Cat. # 62409-002) were coated with the H3 protein, at 1 μg/mL in PBS (Sigma, Cat. # D8537) at 4 °C overnight. Plates were washed 3 times with PBS-Tween 0.1% (Thermo Scientific, Cat. # IBB-170) before blocking with 1% BSA (Thermal Scientific Cat. # 37525) in PBS-Tween 0.1% for 1 h at room temperature. A 3-fold serial dilution with 8 points, starting at an initial dilution of 1:100 was performed for each serum. Plates were washed 3 times after 1 h incubation at room temperature before adding 50 *μ*L of 1:8000 Goat Anti-Mouse IgG H&L HRP (Abcam, Cat. # 6789) to each well. Incubation at room temperature followed for 1 h and plates were washed 3 times. Plates were developed using Pierce 1-Step Ultra TMB-ELISA Substrate Solution (Thermo Scientific, Cat. #: 34028) for 0.1 h and stopped by Pierce stop solution (Thermo Scientific, Cat. #: N600). Plates were read at 450 nm in a Cytation7 reader. Antibody titers were reported as the lowest antibody concentration resulting in an optical density >0.2.

## 3 Results

### 3.1 Spatial transcriptomics reveal temporally and regionally distinct immune activation in draining lymph nodes

To define early spatiotemporal immune responses to vaccination, mice were immunized intramuscularly with an adjuvanted rHA-protein vaccine or PBS control in a prime-boost regimen spaced 21 days apart (**Figure 1a**). Muscle and draining lymph nodes were collected at 24 h (Day 1) and 7 days (Day 7) post-prime for spatial transcriptomic profiling using the GeoMx™ DSP WTA assay and CosMx™ SMI 1k RNA Panel assay. For the GeoMx™ DSP assay, follicular ROIs were chosen to distinguish immune profiles where GC associated B cell responses are expected to develop, from those occurring in surrounding non-follicular lymph node ROIs, where early innate activation, antigen-presenting cell activity, T cell priming, stromal signaling and lymphatic/subcapsular sinus-associated responses are to be detected.

**Figure 2:**
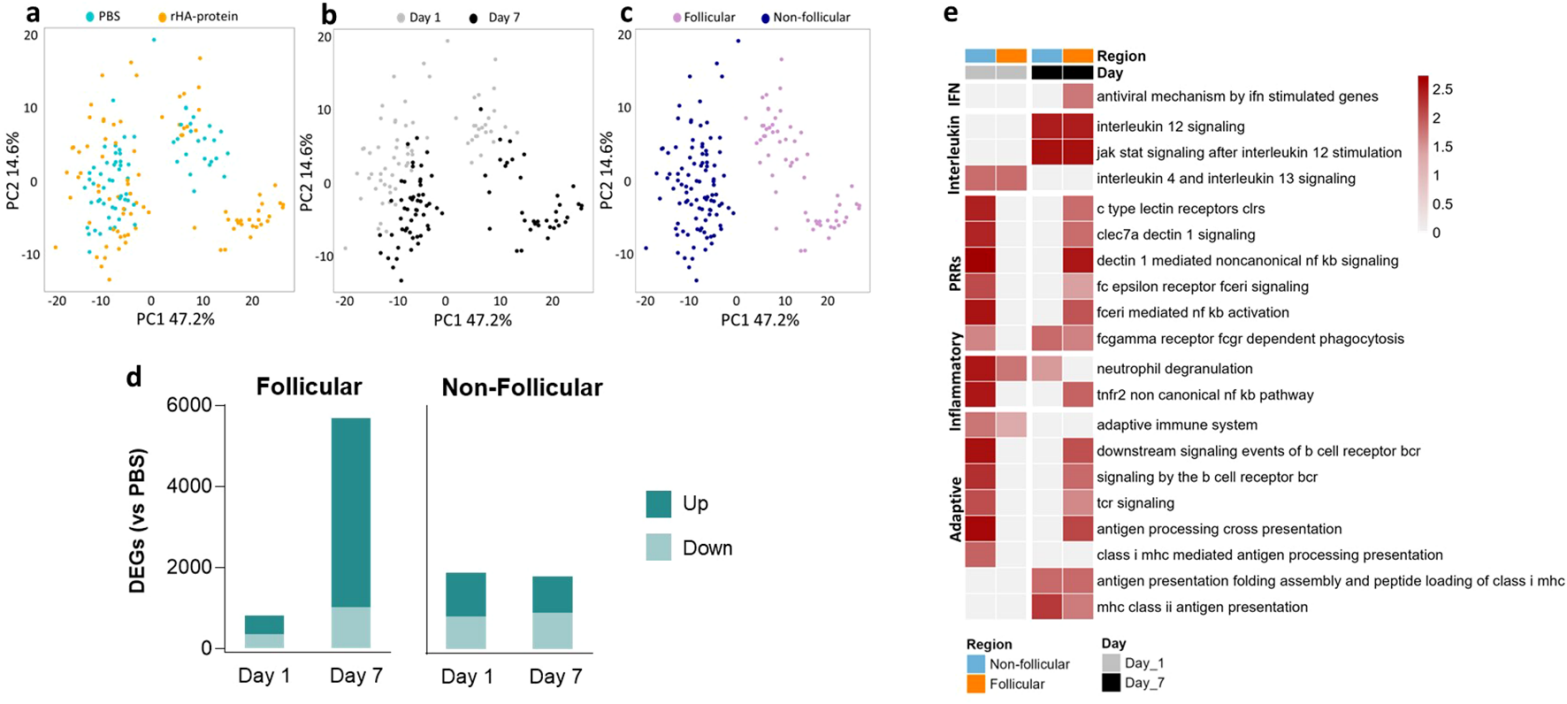
GeoMx™ DSP spatial transcriptional signatures in follicular and non-follicular non-segmented ROIs from lymph nodes following PBS and rHA-protein influenza vaccination. **(a-c)** PCA plots of transcriptomic changes across all non-segmented ROIs. Each dot represents a non-segmented ROI. **(a)** comparison of treatment groups across both time points. **(b)** comparison of time points across all treatment groups. **(c)** comparison of non-segmented ROI region across all treatment groups and timepoints. **(d)** number of DEGs (DEGs) in the adjuvanted rHA-protein vaccine relative to the PBS control group, DEGs threshold of FDR < 0.05. **(e)** REACTOME signaling pathways (FDR < 0.05), normalised enrichment score for in adjuvanted rHA-protein relative to PBS after day 1 and day 7 of prime vaccination.

Principal component analysis (PCA) of lymph node ROIs showed modest segregation by treatment and time point (**Figures 2a, b**). However, the dominant source of transcriptional variation was anatomical region, with follicular and non-follicular ROIs separating strongly along PC1 (**Figure 2c**). Within the follicular compartment, Day 7 ROIs from adjuvanted rHA-protein treated mice formed a more distinct transcriptional grouping along the PC2 axis relative to PBS controls (**Figures 2a-c**). PCA analyses performed separately within follicular and non-follicular ROIs demonstrated that transcriptional variation associated with treatment and timepoint was more readily resolved once the dominant compartmental signal was removed (**Supplementary Figures 2a-d**). In follicular ROIs alone, Day 7 adjuvanted rHA-protein samples showed greater separation from PBS controls, consistent with distinct adaptive immune activation within GC-associated regions (**Supplementary Figure 2a**). In contrast, some separation between PBS and adjuvanted rHA-protein samples was also observed within non-follicular regions (**Supplementary Figure 2c**), the most distinct and biologically interpretable vaccine-associated clustering occurred in Day 7 follicular ROIs. Differential expression analysis identified a >10-fold increase in DEGs in follicular ROIs at Day 7 compared with PBS (**Figure 2d**), enriched for genes linked to GC formation, transcriptional regulation, and immune signaling, supporting the initiation of antigen-specific B cell responses and GC formation (**Supplementary Figure 3**). These large transcriptional changes likely reflect both shifts in cellular composition, including expansion of proliferating GC-associated populations, and cell-intrinsic transcriptional regulation, however, as GeoMx™ DSP profiles whole ROIs as bulk sequencing, single cell spatial transcriptomics sequencing using the CosMx™ SMI was used to directly assess cell type proportional changes (described below).

To further investigate the DEGs, gene set enrichment analysis (GSEA) was run for the adjuvanted rHA-protein relative to the PBS control, which revealed strong innate and adaptive immune pathway activation in the non-follicular regions of adjuvanted rHA-protein–treated mice at Day 1, with significant enrichment of pattern recognition receptor pathways (PRRs) such as C-type lectin receptors, Fc receptor signaling, neutrophil degranulation, IL-4/IL-13 cytokine signaling pathways and adaptive pathways such as B cell receptor (BCR), T cell receptor (TCR) signaling and antigen presentation (**Figure 2e**). Follicular regions remained largely transcriptionally quiescent at this early timepoint, however by Day 7 there was an enrichment of adaptive immune pathways and IL-12 signaling as well as the associated Jak-Stat signaling in both the follicular and non-follicular regions for the adjuvanted rHA-protein. Furthermore, the PRR pathway activity shifted from the non-follicular region at Day 1 to the follicular region at Day 7 for the adjuvanted rHA-protein, with GC-associated genes *Bcl6* and *Aicda* upregulated in rHA-protein lymph-node tissue (**Supplementary Figure 3**). Systemic cytokine measurements revealed a strong early inflammatory response at 6 h post-vaccination that declined by Day 3 (**Supplementary Figure 4a**). The adjuvanted rHA-protein vaccine induced significantly elevated levels of G-CSF, IL-6, IL-13, KC, and MCP-1 at 6 h, which diminished over time. In contrast, these cytokines, apart from G-CSF, were not significantly elevated in PBS treated controls (**Supplementary Figure 4b**). Consistent with these early innate and spatial transcriptional responses, robust HA-specific IgG induction following vaccination was observed (**Supplementary Figure 5**). At Day 14, the adjuvanted rHA-protein vaccine elicited ~3-log higher IgG titers compared with PBS controls, and by Day 35 (post-boost) titers increased by a further ~0.5 log. Collectively, these results indicate a time-dependent shift in immune activation from non-follicular to follicular regions, coinciding with systemic cytokine induction and robust humoral immunity.

### 3.2 Single-cell spatial transcriptomics confirms GC organization and cellular dynamics in adjuvanted rHA-protein lymph nodes

**Figure 3:**
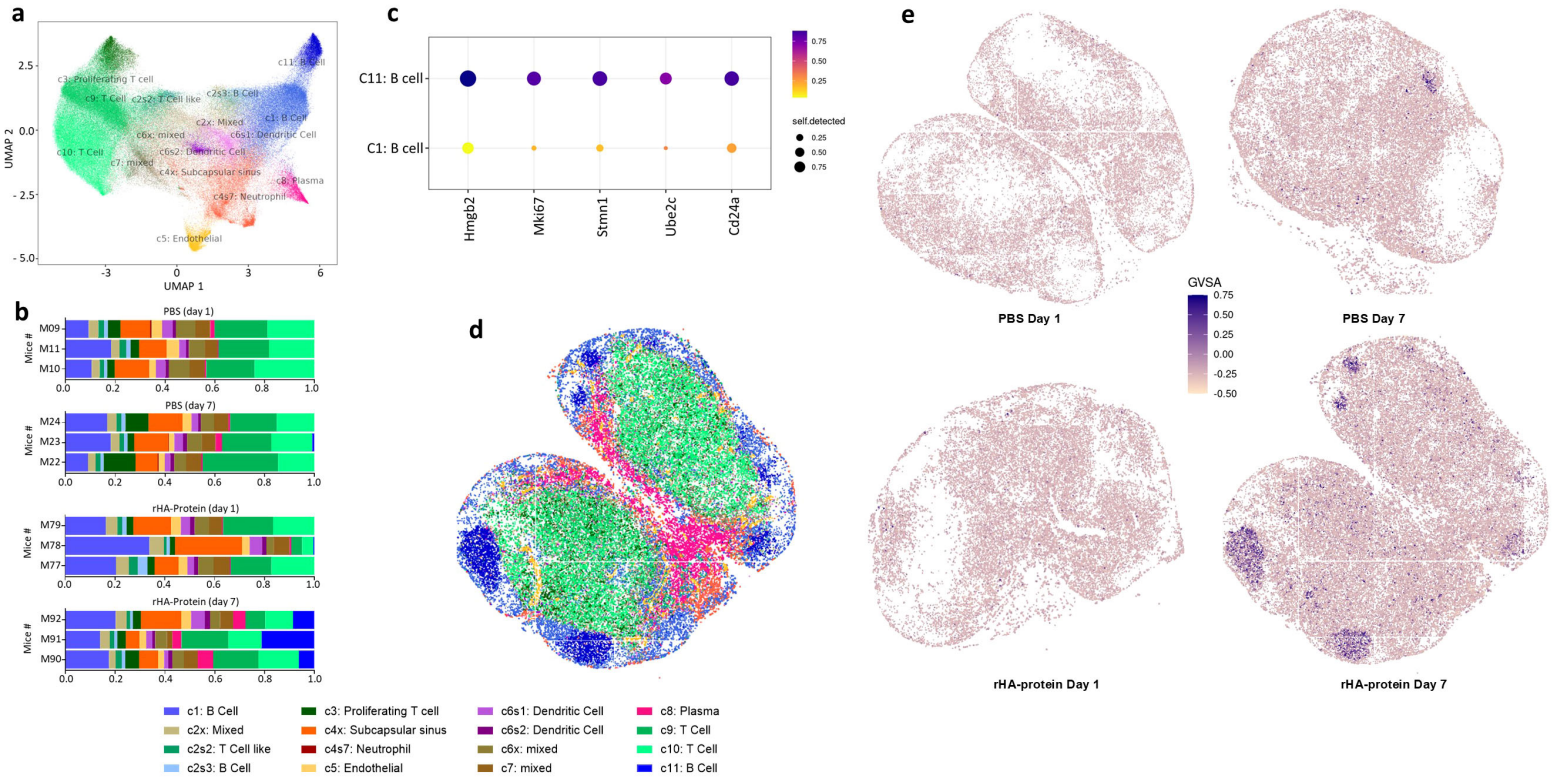
CosMx™ SMI single-cell spatial transcriptomics in lymph nodes following PBS and rHA-protein influenza vaccination. **(a)** Uniform Manifold Approximation and Projection (UMAP) and graph-based clustering identified sixteen clusters representing major immune and stromal cell types. **(b)** Inference of cell type proportions from lymph node single cell spatial transcriptomic data. Each bar in the stacked bar plot represents a lymph node tissue sample for individual mice. Mice numbers are represented to the left of the bar. Bar plots are separated into treatment groups and time points. **(c)** gene expression of genes associated with cell cycle progression and germinal center B cell proliferation as seen in the c1 and c11 B cell cluster. **(d)** spatial mapping of cell types in a lymph node tissue section for the rHA-protein at Day 7. **(e)** spatial mapping of the cell cycle immune pathway in both the PBS and rHA-protein treatment group at Day 1 and Day 7.

To resolve cellular composition and spatial transcriptional heterogeneity at single-cell resolution, ~379,000 cells from all lymph node tissue sections across treatment groups and time points were profiled using CosMx™ SMI. Uniform Manifold Approximation and Projection (UMAP) and graph-based clustering identified sixteen clusters representing major immune and stromal cell types, including B and T cells, DCs, endothelial cells, neutrophils, and subcapsular sinus macrophages (**Figure 3a** and **Supplementary Figure 6** - separated by treatment and time). Dot plots showing top 5 genes for each cluster are shown in **Supplementary Figure 7**.

Among treatment-associated changes, the c11 B-cell cluster showed the strongest enrichment in the adjuvanted rHA-protein Day 7 treatment group relative to PBS control across both time points (**Figure 3b**, **Supplementary Figure 8**). The c8 plasma-cell cluster was enriched in the adjuvanted rHA-protein Day 7 group, indicating a consistent expansion of B cell subsets in response to adjuvanted rHA-protein vaccination, whereas the neutrophil population declined over time (**Figure 3b**, **Supplementary Figure 8**). To distinguish between the two major B cell clusters, c1 and c11, differential gene expression analysis was performed (DEGs tabulated in **Supplementary Table 1**). Cluster c11 exhibited a higher number of genes with positive fold changes compared to c1, suggesting a more activated and proliferative B cell state. Notably, genes such as *Hmgb2*, *Mki67*, *Stmn1*, and *Ube2c*, which are associated with cell cycle progression and proliferative GC-like B cell states, were upregulated in c11 (**Figure 3c**). Additionally, *Cd24a*, a marker of early B cell development, was significantly elevated in c11. In contrast, *Sell* (*Cd62L*), *Cd55*, and *Bcl2*, genes typically linked to resting, long-lived, or memory B cells, were downregulated in c11. The c11 cluster was predominantly observed within follicular regions of the lymph node at Day 7 following adjuvanted rHA-protein treatment (**Figure 3d**). To quantitatively assess the proliferative state of the B cell populations, FGSEA pathway enrichment analysis was performed between the c11 and c1 B cell clusters. This demonstrated significant enrichment of cell cycle associated immune pathway within the c11 cluster relative to c1 (adjusted *p* value = 1.8 × 10^−5^), consistent with a highly proliferative GC-like B cell phenotype (FGSEA results for the c11 versus the c1 B cell cluster tabulated in **Supplementary Table 2**). To illustrate the spatial distribution of proliferative activity, GSVA scores for the cell cycle immune pathway were mapped onto the lymph node tissue sections. Elevated cell cycle immune pathway activity was predominantly localized to follicular regions at Day 7 in the adjuvanted rHA-protein treatment group (**Figure 3e**). In contrast, within the PBS control group, elevated cell cycle pathway activity was detected only in a small number of spatially restricted regions, which were primarily localized outside follicular areas, with only a small focal area of activity observed adjacent to follicular regions (**Figure 3e**). Together, these data indicate that adjuvanted rHA-protein vaccination drives a spatially coordinated, follicular-localized proliferative B cell response within the first week post-vaccination.

### 3.3 Injection site spatial transcriptomics highlights early fibroblast and muscle cell activation in adjuvanted rHA-protein–vaccinated muscle

In addition to immune spatiotemporal changes in the lymph node, we also looked at upstream immune activation in muscle tissue of the injection site. Because robust cell segmentation could not be achieved in injection site muscle tissue, CosMx™ SMI data from these samples were analyzed using 20×20 µm spatial grids rather than segmented single cells. Accordingly, the clusters described below represent transcriptionally defined spatial grid regions enriched for muscle, fibroblast, adipocyte or immune associated profiles, rather than discrete cell populations.

**Figure 4:**
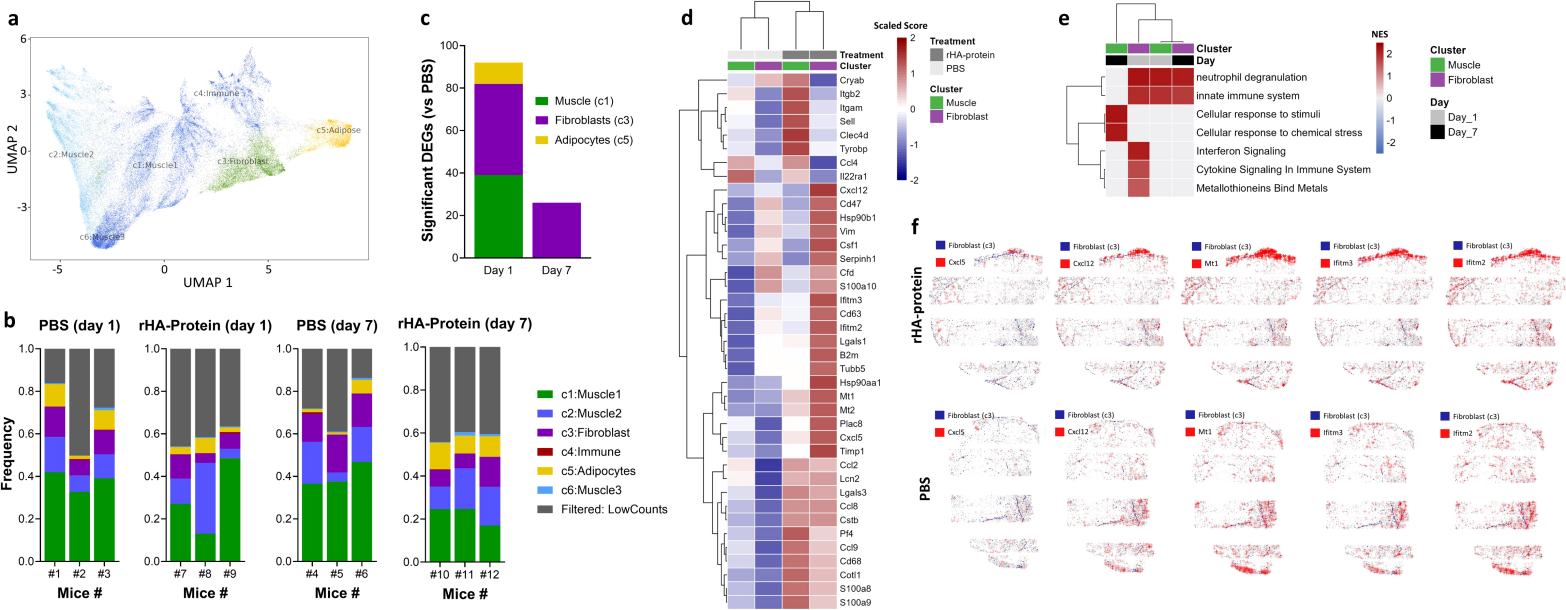
CosMx™ SMI Single-cell spatial transcriptomics in muscle tissue at site of injection following adjuvanted rHA-protein influenza vaccination. **(a)** Uniform Manifold Approximation and Projection (UMAP) and graph-based clustering identified six clusters representing major immune and stromal cell types. **(b)** Inference of enriched cluster proportions from muscle tissue spatial transcriptomic data. Each bar in the stacked bar plot represents a muscle tissue sample for individual mice. Mice numbers are represented to the left of the bar. Bar plots are separated into treatment groups and time points. **(c)** Number of DEGs for each enriched cluster for each treatment group contrast at each time point. DEGs threshold of FDR < 0.05. **(d)** Scaled expression values for immune related genes at Day 1 in fibroblast and muscle enriched clusters for each treatment group. **(e)** REACTOME early immune signaling pathways (FDR < 0.05) showing normalised enrichment score for rHA-protein relative to PBS after day 1 in fibroblast and muscle enriched clusters. **(f)** Spatial mapping of immune related genes in muscle tissue section for the rHA-protein and PBS at Day 1.

A top-level clustering identified 6 spatial grid clusters, with 3 of these assigned as muscle enriched clusters (c1, c2 and c6). Fibroblasts enriched clusters (c3), adipocytes enriched clusters (c5) and a small immune associated cluster (c4) were also identified (**Figure 4a**, **Supplementary Figure 9** - separated by treatment and time). Dot plots showing top 5 genes for each cluster are shown in **Supplementary Figure 10**. Across Day 1 and Day 7 post-treatment, most spatial grids were assigned to muscle enriched clusters, particularly c1 and c2, and to a lesser extent c6, indicating that muscle associated transcriptional regions remained the dominant spatial feature of the injection site tissue (**Figure 4b**). Adipocyte abundance transcripts varied between mice and treatments; however, this likely reflects natural variability in fat deposition at the injection site rather than a vaccine-specific effect. Similarly, differences in fibroblast transcript proportions across groups and time points may stem from individual differences in connective tissue content within the muscle rather than being driven by treatment. In addition to this, immune cell infiltration was minimal across all groups and time points.

Muscle enriched and fibroblasts enriched clusters had the highest number of DEGs in the adjuvanted rHA-protein treatment group, relative to PBS control, at Day 1 (**Figure 4c**). By Day 7, DEG counts were reduced, with the fibroblast enriched cluster showing the most persistent transcriptional changes. Most top DEGs in Day 1 fibroblast and muscle enriched clusters were immune-related (**Figure 4d**). In contrast, PBS controls showed only mild interferon-stimulated gene (ISG) up-regulation, including *Ifitm2* and *B2m*, indicating a transient, low-level innate response. To functionally interpret the top DEGs identified in muscle and fibroblast enriched clusters at Day 1, we performed GSEA using top immune-related gene sets (**Figure 4e**). Although the CosMx™ SMI platform is limited to 1,000 probes, the significantly upregulated genes in adjuvanted rHA-protein treated tissues relative to PBS control at Day 1 aligned with key innate immune pathways. For fibroblast and muscle enriched clusters this included neutrophil degranulation (adjusted *p* values < 2.8 × 10^−5^ and normalized enrichment score (NES) > 2.2) and innate immune system pathways (adjusted *p* values < 1.5 × 10^−5^ and NES = 2.1) and cytokine signaling in the immune system (adjusted *p* values = 1.2 × 10^−4^ and NES = 1.7) were pathways upregulated in fibroblast enriched clusters in the adjuvanted rHA-protein-treatment group relative to PBS at Day 1.

Based on these findings, we next spatially mapped some key immune related genes, *Cxcl5, Cxcl12, Ifitm2, Ifitm3* and *Mt1*, within the Day 1 injection site tissue sections for both the adjuvanted rHA-protein treatment group and PBS controls (**Figure 4f**). Indeed, these genes were significantly upregulated in the fibroblast enriched cluster at Day 1 in the adjuvanted rHA-protein treatment group relative to PBS controls, with adjusted *p* values < 7 × 10^−4^ and log_2_ fold changes > 1.4 (DEGs tabulated in **Supplementary Table 3**). From the differential expression analysis, the expression of these genes was also substantially lower at Day 7 relative to Day 1 in fibroblast enriched cluster from the adjuvanted rHA-protein treatment group (adjusted *p* values < 1.1 × 10^−4^ and log_2_ fold changes > 1.6), whereas no significant temporal differences were observed in PBS controls.

## 4 Discussion

Here, using GeoMx™ DSP and CosMx™ SMI we demonstrate that an adjuvanted rHA-protein vaccine elicits spatiotemporally distinct immune profiles, particularly in terms of innate immune activation, lymph node compartmentalization and potential GC formation. Pathways activated and locally concentrated genes at the site of injection, particularly chemokine and cytokine signaling in fibroblasts and muscle enriched clusters, were associated with lymph node responses. Here, this early local inflammation at Day 1 corresponded to innate pathway activation in non-follicular regions and later to follicular pathway enrichment associated with GC-like activity at Day 7. Together, these findings provide a previously unresolved view of what, when and where local inflammation and lymph node organization are spatially and temporally associated induced by an AF03 adjuvanted rHA-protein vaccination.

The observed up-regulation of interferon, chemokine/cytokine and myeloid-activation genes (Ifitm2/3, *Cxcl5*, *Cxcl2*, *Ccl2*, *Ccl8*, *Cd68*, *Cd63*, *Mt1/2*, *S100a8/9*) seen in the muscle aligns with known early effects of oil-in-water emulsion adjuvants at the injection site (2, 12, 27). In the case of the oil-in-water emulsion adjuvant AS03, which contains squalene, polysorbate 80 and α-tocopherol, the early immune events at the injection site have been attributed to the α-tocopherol component. α-Tocopherol has been shown to modulate the expression of chemokines and cytokines, including CCL2, CCL3, IL-6, G-CSF/CSF3 and CXCL1, enhance antigen uptake by monocytes and promote granulocyte recruitment to draining lymph nodes (13). In the case of AF03, which is less well characterized in terms of early immune events, does not contain α-tocopherol but contains pan 80, EmulginB1 and mannitol instead. The other well characterized oil-in-water emulsion adjuvant, MF59, made up of squalene, polysorbate 80 and Span 85, has been shown to activate innate immune cells, including macrophages and dendritic cells as well as promote production of chemokines such as CCL2, CCL4, CCL5 and CXCL8 (2, 9, 28, 29). The findings presented here extend the resolution of immune responses induced by adjuvanted protein vaccines by spatially mapping chemokine and interferon-stimulated gene (ISG) induction to fibroblast enriched clusters at the injection site, revealing a microanatomical immune transcriptomic landscape that has not previously been described for an oil-in-water emulsion-adjuvanted recombinant protein vaccine. Whether this architecture is unique to AF03 adjuvanted rHA-protein vaccines or reflects a broader organizing principle across effective other squalene-based oil-in-water emulsion adjuvants or other vaccine platforms remains to be determined.

The transient kinetics of the cytokine/chemokine immune profiles at the injection site are consistent with oil-in-water emulsion adjuvant, AS03, which induces transient cytokine and chemokine activity in muscle within the first 6–24 hours after vaccination, supporting rapid immune-cell recruitment (13). The limited and potentially unexpected immune cell infiltration observed at the injection site may partly reflect the timing at Day 1. MF59 adjuvant has been shown to induce rapid recruitment of immune cells to injected muscle in the first 6 h post-vaccination with neutrophil concentration peaking at 16 h and the declining with time. Similarly, with alum-adjuvant intramuscular injection, immune cell infiltrate was detected at the injection site as early as 2–6 h after vaccination, with an initial neutrophil dominated response followed by macrophages, eosinophils and MHC-II^+^ cells peaking between Day 2 and Day 7 post vaccination (30). Therefore, analyses at Day 1 may not have captured the peak or full temporal range of local cellular infiltration, particularly if immune cell recruitment was transient, cell type specific or spatially focal within the injected muscle.

This adjuvant-induced response was associated with upregulation of C-type lectin receptor and Fc receptor pathways, alongside cytokine and inflammatory signaling pathways at Day 1 in the non-follicular region of the lymph node. Oil-in-water emulsion adjuvants, such as AS03 and MF59, are typically described as engaging Nod like receptor protein 3 (NLRP3)-independent antibody secreting cell activation and Toll-like receptor (TLR)-independent MyD88 pathways (12, 31). From the GeoMx™ DSP data here, it is possible that immune cascades triggered by early events, including immune complex formation, cell death and debris as well as chemokine release, contributed to a later wave of PRR pathway activation or represents secondary activation of dendritic cells at 24 hours and even at Day 7 post-vaccination. Indeed, in mouse lymph nodes, AS03 can induce gene expression changes, particularly in cytokine-cytokine receptor interaction pathways, as early as 2–6 hours post-vaccination (32). Furthermore, AS03 components have been shown to drain to the lymph node in as little as 30 minutes (33). Early responses include transient downregulation of lipid metabolism-related genes at 2 hours and strong upregulation of cytokine interaction, MAPK, and STAT signaling pathways by 6 hours (32). In addition to PRR pathway upregulation at Day 7, follicular regions from the adjuvanted rHA-protein group exhibited enrichment of IL-12 and JAK/STAT signaling from the GeoMX™ DSP data, coinciding with proliferative B cell expansion and transcriptional signatures of GC formation. IL-12 and IL-6 are key cytokines linking innate inflammation to adaptive lymphocyte responses, including STAT-dependent induction of *Bcl6*, Tfh differentiation and downstream GC development (34, 35). Consistent with this pathway, *Bcl6* and the GC-associated gene *Aicda* were upregulated in rHA-protein lymph-node tissue, supporting the presence of an early GC-associated transcriptional profile. CosMx™ SMI data collected across Day 1 and Day 7 supported the emergence of an expanded, organized and proliferative c11 B cell cluster within follicles, consistent with a GC-like phenotype rather than the diffuse or extrafollicular B cell activation observed in PBS controls. This pattern is consistent with a predominantly follicular B cell response and contrasts with the prominent extrafollicular B cell responses reported in settings of sustained TLR4 agonism, where prolonged innate inflammatory signaling drives rapid antibody-secreting cell differentiation independently of germinal center formation (36).

## 5 Limitations

Day 1 and Day 7 were selected to capture the transition from early innate activation at the injection site (Day 1) to the emergence of adaptive immune responses, including GC formation, in the draining lymph node (Day 7). Yet, inclusion of post-boost or later timepoints would further enable characterization of GC maturation, memory formation, and recall responses. Also, this study did not include an AF03-only control. The inclusion of such a control would enable clearer delineation of the relative contributions of the adjuvant and antigen to the observed injection site responses. Multiple ROIs were collected from each tissue section to capture regional variation, represent the lymph node microanatomical compartments under study, particularly follicular and non-follicular regions and reduce the impact of individual ROIs failing sequencing or not meeting quality control thresholds, however, the relatively modest sample size of n = 3 mice per group/timepoint limits statistical power and may reduce the ability to capture biological variability between animals. Furthermore, the CosMx™ SMI analysis was performed using a targeted 1000-plex mouse panel which, although enabling spatial profiling across a broad range of immune and stromal transcripts, does not comprehensively capture the full transcriptome and may therefore omit additional vaccine-relevant genes and pathways. Importantly, several canonical GC markers, including *Bcl6*, *Aicda*, *S1pr2* and *Gcsam*, were not represented in this panel, limiting the ability in definitively assigning GC B cell identity and resolving distinct GC profiles, including light-zone and dark-zone associated states, at single-cell resolution.

## 6 Conclusion

Overall, by defining both local and downstream immune responses at cellular and spatial resolution, this study provides a previously unresolved view of what, when and where immune responses induced by an AF03 adjuvanted rHA protein vaccine occur across the injection site and draining lymph node during the first week following vaccination. The ability to relate these architectural changes to adjuvant-driven innate cues and downstream measures adds an additional level of resolution for understanding vaccine efficacy. Future studies incorporating approaches such as knockout mouse models or *in vitro* perturbation experiments could be used to establish mechanistic links between immune events at the injection site and the draining lymph node across a range of settings, including immunosenescence, alternative routes of vaccine delivery and repeated vaccination.

## Data Availability

The GeoMx™ DSP lymph node dataset presented in this study is deposited in the GEO repository, accession number GSE328253. The CosMx™ SMI lymph node and muscle dataset presented in this study is deposited in the GEO repository, accession number GSE336618.

## Supplementary material

Supplementary Figure 1 Scanned images for spatial transcriptomics. Hematoxylin and eosin-stained (H&E) and digitally scanned representative images of **(a)** lymph node tissue section and **(b)** injection muscle tissue section. **(c)** Scanned image of lymph node tissue section with segmentation using Cyto2 model from CosMx SMI run (left). Cell type labels for each segmented cell (right). **(d)** Scanned image of muscle tissue section from CosMx SMI run (left). Due to difficulty acquiring acceptable cell segmentation on the muscle samples, the muscle samples were gridded into 20x20 µM grid squares rather than true cells. Cell types called for each grid (right).

Supplementary Figure 2 PCA plots of GeoMx DSP transcriptomic changes across all non-segmented ROIs, separated by follicular and non-follicular region. **(a-d)** each dot represents a non-segmented ROI. **(a)** comparison of treatment groups across both time points for follicular region. **(b)** comparison of time points across all treatment groups for follicular region. c, comparison of treatment groups across both time points for non-follicular region. d, comparison of time points across all treatment groups for non-follicular region.

Supplementary Figure 3 Key genes associated with, or products of, germinal center development identified as contributors to the Day 7 follicular rHA-protein cluster in the GeoMx™ DSP data. Scaled normalised expression values for these germinal center related genes in the Day 7 follicular rHA-protein cluster were compared against Day 1 follicular rHA-protein, as well as Day 7 and Day 1 follicular regions from the PBS treatment group.

Supplementary Figure 4 Dynamics of serum cytokine levels after vaccination. Serum cytokine responses were measured at 0, 6, 24, 72 (3 days) and 168 h (7 days) post vaccination in mice receiving either **(a)** adjuvanted rHA-protein vaccine or **(b)** PBS control. Statistical significance was assessed using one-way ANOVA with Dunnett’s multiple comparisons test, comparing each time point to the 0 h baseline. *P<0.05, **P<0.01, ***P<0.001, ****P<0.00001.

Supplementary Figure 5 Serum IgG titres measured by ELISA following vaccination. IgG antibody responses were quantified in serum collected at Day 14 (post-prime) and Day 35 (post-boost) from mice receiving either PBS control or adjuvanted rHA-protein vaccine. Bars show the geometric mean IgG titres for each group, with error bars representing the geometric standard deviation.

Supplementary Figure 6 Uniform manifold approximation and projection (UMAP) representation of lymph node single cell CosMX™ SMI spatial transcriptomic data annotated with mouse identification number. Data set comprised all lymph node tissue sections. UMAPs separated by treatment group and time point.

Supplementary Figure 7 Dot plot of top 5 genes for each lymph node tissue cluster from the CosMX™ SMI spatial transcriptomic data. Sixteen clusters representing immune cell types were identified in the lymph node tissue sections. Data was subsampled to 1000 cells per cluster for marker gene calculation. Size of dot indicates proportion of cells within group expressing the gene.

Supplementary Figure 8 Significant cell type proportion changes in the adjuvanted rHA-protein treatment relative to the PBS control in the CosMx™ SMI data. The cell types that changed significantly (FDR < 0.05) at each time point for the rHA-protein treatment relative to the PBS control are colored according to the fold change. Red and blue indicate a positive and negative fold change respectively.

Supplementary Figure 9 Uniform manifold approximation and projection (UMAP) representation of site of injection CosMX™ SMI spatial transcriptomic data annotated with mouse identification number. Data set comprised all site of injection tissue sections. UMAPs separated by treatment group and time point.

Supplementary Figure 10 Dot plot of top 5 genes for each site of injection tissue cluster from the CosMX™ SMI spatial transcriptomic data. Six clusters representing immune cell types were identified in the site of injection tissue sections. Data was subsampled to 1000 cells per cluster for marker gene calculation. Size of dot indicates proportion of cells within group expressing the gene.
